# Scope of Nursing Care in Polish Intensive Care Units

**DOI:** 10.1155/2013/463153

**Published:** 2013-12-31

**Authors:** Mariusz Wysokiński, Anna Ksykiewicz-Dorota, Wiesław Fidecki

**Affiliations:** ^1^Chair of Development in Nursing, Faculty of Nursing and Health Sciences, Medical University, U1. Staszica 4-6, 20-081 Lublin, Poland; ^2^Chair and Department of Management in Nursing, Faculty of Nursing and Health Sciences, Medical University, Lublin, Poland

## Abstract

*Introduction*. The TISS-28 scale, which may be used for nursing staff scheduling in ICU, does not reflect the complete scope of nursing resulting from varied cultural and organizational conditions of individual systems of health care. *Aim*. The objective of the study was an attempt to provide an answer to the question what scope of nursing care provided by Polish nurses in ICU does the TISS-28 scale reflect? *Material and Methods*. The methods of working time measurement were used in the study. For the needs of the study, 252 hours of continuous observation (day-long observation) and 3.697 time-schedule measurements were carried out. *Results*. The total nursing time was 4125.79 min. (68.76 hours), that is, 60.15% of the total working time of Polish nurses during the period analyzed. Based on the median test, the difference was observed on the level of *χ* ^2^ = 16945.8,*P* < 0.001 between the nurses' workload resulting from performance of activities qualified into the TISS-28 scale and load resulting from performance of interventions within the scopes of care not considered in this scale in Polish ICUs. *Conclusions*. The original version of the TISS-28 scale does not fully reflect the workload among Polish nurses employed in ICUs.

## 1. Introduction

Considering the growing problem of the shortage of nursing staff which has been observed in Poland, the determination of optimum standards for nursing staff scheduling in individual wards has become an urgent problem. Staff scheduling is one of the elements of work standardization. In Poland, this concept is understood as “an established size of the work group creating one team (…), which is assigned specified duties in performing necessary activities (…) stipulated in the work regulations at an individual workplace or ward (…)” [1]. Therefore, nursing staff scheduling should be based on the determination of the number and occupational structure of nurses, indispensable for optimum performance of tasks within the given planning period [2]. The number of staff necessary for performing individual jobs in specified technical, economic, and organizational conditions, with the consideration of demands posed by psychology, ergonomics, and work safety, may be determined by means of analytical and summary methods [1]. Summary methods specify work standards by estimation, without former analysis of work methods, conditions, and allocation of work between smaller components. Their characteristic feature is that they fix the actual state of the organization and do not mobilize changes and activities on behalf of improvement of the quality of care provided. Analytical methods, however, require an earlier performance of a critical analysis of methods and work conditions, their improvement, division of work into smaller elements, and only then a later application of research methods derived from the observation of the course of standardized work [3]. The measurement techniques used within the analytical method are as follows: day-long observation of the workday, continuous observation, time scheduling, and snap-shot observation. In these techniques, it is most important to determine the duration of individual components contributing to the task. In the case of nursing workplaces, analytical methods are the most reliable and close to reality.

This is especially important in the case of wards generating high costs of functioning, for example, intensive care units. Until 1999 in Poland, nursing staff scheduling in health care facilities resulted from the number of hospital beds. This method did not allow the optimum use of human resources. In this method, job positions were ascribed to beds, without consideration of the percentage rate of their use, actual demand for care, organizational-technical conditions in a given hospital, and the time at the disposal as worked out by nurses [4]. Attempts were undertaken to change this situation by introducing in 1999 the regulation in the matter of minimum standards for employment of nurses and midwives [5]. However, all of the responsibility for the development of these standards was ceded to the nursing staff, at that time unprepared for tasks related to the standardization of work. It was decided to use the technique of self-observation as the main source of data concerning nursing care and the possibility to determine standards individually by particular health care facilities. This leads to the situation that wards located in the same area and providing a similar scope of health services could have various nurse staffing standards.

Therefore, various methods are applied using, among other things, the calculation of patients' demand for care based on the qualification of patients in specified categories. Among these methods is the TISS-28 scale which, in the case of Polish ICUs, is commonly used for the settlement of accounts with the payer—the National Health Insurance Agency. Apart from the abovementioned use, the scale may also be applied for the assessment of the severity of patients' status, selection of the optimum place of treatment, estimation of employment standards, determination of an optimum number of nurses, and evaluation of loading them with work for the needs of anesthesiology and intensive care units [6–12]. This scale allows an objective assessment of the use of therapeutic means and intensification of the therapeutic activities, and consequently, the contribution of work, including that of nurses. Therefore, it also reflects patient demand for nursing care. The TISS was first applied in 1974 [6, 13, 14]. In subsequent years, this scale was modified and functioned as TISS-28 scale. The studies confirmed a high correlation between its versions [15]. A great advantage of the TISS-28 is the easiness of collecting data, because it requires devoting less than 5 minutes/patient/day [16]. At the same time, the evaluation of the state of a patient by the TISS scale should be performed once every 24 hours at the same hour, for example, at 08.00. Nevertheless, due to frequent changes in the intensity of the work of nurses in intensive care units, some researchers in their studies performed the determinations by the TISS-28 scale during each 8-hour shift [17, 18].

The scale consists of seven criteria associated with therapeutic intervention: basic care procedures, respiratory therapy, cardiovascular therapy, treatment supporting renal excretory function, monitoring of the CNS function, treatment of metabolic disorders, and other interventions [13, 19]. A proper number of scores from 5 to 1 are ascribed to the criteria. The most advanced interventions, performed in patients in most severe state, are ascribed 5 scores, whereas routine interventions—1 score. Within each criterion are classified interventions which refer to this criterion.

In consideration of the above, in Poland, attempts are increasingly more often undertaken to apply this method also for the calculation of nursing services provided in this type of unit. However, it should be kept in mind that the scale was created in different cultural and organizational conditions and cannot fully reflect the activities undertaken by Polish nurses in intensive care units. This especially refers to the nursing time, which may depend on many factors, for example, type of facility, unit, provision with medical equipment and medical instruments, scope and of activities of the auxiliary services, but primarily on the competence and qualifications of the staff and adopted nursing care standards and procedures [4]. This scale focuses mainly on therapeutic interventions associated with the performance by nurses of the therapeutic-diagnostic function. This may also result from the fact that in other systems of health care there is a lower level nursing staff called nurse assistants, or nursing assistants, whose task is mainly the performance of care and nursing activities. Unfortunately, in Poland, this type of the nursing staff does not exist. This fact may be the reason for which the TISS-28 scale does not fully reflect the engagement of nurses in the care of patients.

The majority of interventions mentioned in the original TISS-28 scale are among the activities which a Polish nurse performs on a doctor's order or only assists in their performance. These interventions are mainly focused on activities resulting from the severity of a patient's condition. An attempt to ascribe the nursing activities observed to the individual scores in the TISS-28 scale carries the risk of various interpretations of their qualification. Therefore, the National Health Insurance Agency [20] prepared guidelines in the area of qualification of the activities performed in Polish intensive care units into the TISS-28 scale.

The objective of the study was an attempt to provide a reply to the following question: what scope of nursing care provided by Polish nurses in intensive care units does the TISS-28 scale reflect?

## 2. Material and Methods

The methods for working time measurement were used for the analysis of the nursing time. On the days when the evaluation of patients was performed according to the TISS-28 scale, the analysis of the nursing time was carried out based on research techniques such as continuous observation (day-long observation) and time scheduling of activities. The data for the study was collected by the first and the third authors, possessing Master's degree in nursing, and university lecturers, who were not occupationally related to the facilities where the study was conducted. This allowed the maintenance of objectivity.

At the first stage of the study, 21 continuous observations were carried out of 12-hour day and night duties, that is, the total of 252 hours of observation. During this time, 75 nursing activities were selected performed by Polish nurses in ICU (Table 1). Simultaneously, the duration of these activities was determined by performing time schedule measurements. At this stage of the study, a total number of 3.813 time schedule measurements were carried out of the nursing activities observed. The measurements covered exclusively the activities performed by nurses employed in an individual unit. Out of the total number of measurements, after outlier removal from the time schedule sequence, 3.697 measurements were qualified for further studies.

The second stage of the study was the verification of the affiliation of individual activities to the criteria of the TISS-28 scale based on the guidelines by the National Health Insurance Agency. At the subsequent phase of the study, the duration was determined of all the activities qualified into the criteria of the TISS-28 and then the durations of activities not considered by this scale.

The studies were conducted from the end of June 2005 to the beginning of January 2009 in 6 intensive care units selected at random in the area of south-eastern Poland: the Health Care Facility in Ostrowiec Świętokrzyski, Independent Public Health Care Facility in Łuków, Cardinal Stefan Wyszyński Regional Specialist Hospital in Lublin, John Paul II Regional Sub-Carpathian Hospital in Krosno, Independent Public Clinical Hospital No. 1 in Lublin and the Independent Public Clinical Hospital No. 4 in Lublin. All the wards where the study was conducted were in state institutions. Considering the specificity of the activities undertaken and the costs of functioning in the territory of the study, there were no private intensive care units. In the case of Polish ICUs, they may be classified into three categories, based on the scope of the services provided. In the first category units, basic procedures are performed within the scope of intensive care, most often in association with postoperative conditions (e.g., wards in provincial hospitals). The second category covers wards which possess a wider access to advanced and costly procedures (e.g., regional hospitals), whereas in the third category are classified ICUs, where patients are treated requiring advanced procedures (e.g., clinical hospitals) [21]. This proposal is related to the reference level of hospitals. On the first and second reference level each, two wards were selected at random: ICU at the Health Care Facility in Ostrowiec Świętokrzyski, the Independent Public Health Care Facility in Łuków, and wards at the Cardinal Stefan Wyszyński Regional Hospital in Lublin and John Paul II Regional Sub-Carpathian Hospital in Krosno. The third level of reference is represented by only two university units functioning in this region, that is, ICU within the Independent Public Clinical Hospital No. 1 in Lublin, and the Independent Public Clinical No. 4 also in Lublin. This selection of wards enabled the study to cover the wards providing health services on different levels.

The results obtained were subjected to statistical analysis by means of the software Statistica 6.0. Selection of the statistical methods used in the presented study was conditioned by the scope of data subjected to the analysis. For measurable quantitative traits (e.g., age and day of hospitalization) basic statistical measures were calculated: arithmetic mean, standard deviation, median, and minimum, and maximum values. Median test was applied. 5% error risk was adopted in the study.

Consent was obtained from the managers of the previously mentioned facilities to conduct the studies.

## 3. Results

From among all the 75 nursing activities observed which were performed by nurses during 3.697 time schedule measurements, these lasted jointly for 411581.13 sec (6859.6855 min, i.e., 114.328 hours). Out of this number, 51 activities were qualified as those performed within the TISS-28 scale. Thus, 2.457 time scheduling measurements were used for the analysis. The total nursing time obtained from these measurements was 247547.2 sec (4125.79 min, i.e., 68.76 hours), that is, 60.15% of the total working time of Polish nurses during the period analysed.

During the period examined, Polish nurses devoted a total of 163280.1 sec to the performance of the category “basic procedures” in the TISS-28 scale, which constituted 39.67% of the total time devoted to nursing in the units, where the study was conducted. On average, the performance of the activities within this category occupied 104.53 sec (Me = 42.6, SD = 207.72). Within this category, activities of “administration of a single drug/many drugs” were most frequently performed 593 times and “standard monitoring” 491 times. The nurses employed in the ICUs in the study devoted within nursing the following amount of time to the performance of individual subcategories in the TISS-28 scale: 3.93% of working time to “standard monitoring,” 1.56% to “laboratory tests,” 9.67% to “administration of a single drug/many drugs,” 21.97% to Standard change of dressing”, 2.0% to “frequent change of dressings” and 0.54% to “presence of drainages.”

In the period analysed, the nurses devoted a total of 40627.5 sec to the performance of activities within category 2 “respiratory therapy” in the TISS-28 scale, which constitutes 9.87% of time devoted to nursing. On average, the performance of activities within this category occupied 67.37 sec (Me = 16, SD = 142.91). Considering this category, the procedures within “supporting respiratory system function” were most frequently performed 512 times. The nurses employed in ICUs in the study devoted the following amount of time to the performance of nursing activities within individual subcategories: 0.08% of working time to “mechanical ventilation,” 0.08% to “supporting respiration,” 0.45% to “Care of intratracheal tube or tracheostomy”, and 9.26% to “Supporting respiratory system function.”

According to the guidelines by the National Health Insurance Agency, into category 3 “cardiovascular therapy” only the assisting at procedures and diagnostic tests may be classified The majority of nursing tasks associated with this category are evaluated in category 1, concerning the amount of drugs administered, care of central venous catheters, and changing dressings. For example, the number of vasoactive drugs applied does not lie within the occupational independence of Polish nurses. During the period analyzed, the nurses assisted 31 times at procedures and diagnostic tests. This consumed a total of 10021 sec of their time; on average a single procedure lasted for 323.25 sec (Me = 120, SD = 512.75). The procedures performed within this category constituted 2.44% of the nursing time in the ICUs examined.

During the period of the study, the nurses devoted a total amount of 24981.8 sec to the performance of tasks within category 4 of the TISS-28 scale, which constitutes 6.07% of the nursing time. On average, the performance of the activities within category 4 occupied 114.07 sec (Me = 32, SD = 338.26). Interventions within “measurement of diuresis” were most often performed within category 4–197 times, while the activities within “Hemofiltration, hemodialysis”—were performed 22 times. The nurses employed in the ICUs examined devoted 4.43% of the nursing time to “Hemofiltration, hemodialysis”, and 1.64% to “measurement of diuresis.”

During the study, none of the patients received assistance within the scope of interventions connected with the monitoring of CNS function; no interventions performed by nurses were observed in this area (category 5 of the TISS-28 scale).

During the period analyzed, the nurses devoted 8636.8 sec to the performance of interventions qualified in category 6 “metabolic treatment” of the TISS-28 scale, which constituted 2.1% of the total nursing time. On average, the performance of activities within this category occupied 205.63 sec (Me = 180, SD = 132.07).

Category 7 of the TISS-28 scale covered interventions in the ICUs and outside the units. Within this category the role of a nurse consists mainly of assisting with the performance of these interventions or care of a patient during transportation. In the presented study, this scope of occupational activity was considered in category 3 of the TISS-28 scale.

Thus, the interventions carried out by nurses, classified into the TISS-28 scale, constituted 60.15% of the nursing time. In this area, the tasks associated with basic procedures were the most burdensome, that is, mainly the measurement of patient's life parameters. The remaining 39.85% of time devoted by Polish nurses to the performance of interventions in the ICUs examined was not considered in the original TISS-28 scale.

From among 75 activities performed by the nurses in the study in the ICUs examined, 24 interventions were not considered in the original TISS-28 scale, according to the guidelines by the National Health Insurance Agency. These interventions were grouped into the following scopes of activities: maintaining patency of catheters (covered: flushing of peripheral venous catheter, removal of peripheral venous catheter, and insertion of peripheral venous catheter), assistance to patients with nutrition (feeding a patient, administration of oral fluids, insertion of gastric tube, and removal of gastric tube), assisting patients with excretion (giving a bedpan, removal of urinary catheter, exchange of urine bag, and performing enema), assisting patients with maintaining physical activity (rehabilitation exercises and tilting a patient to erect position), assistance with patient's personal hygiene (hygienic hand washing, handling a strip of lignin, patient's whole body toilet, patient's partial toilet, and change of bed sheets with a patient in bed), assessment of the state of health of a patient (direct observation of a patient, indirect observation of a patient and taking smear for tests), making and maintaining a therapeutic contact with a patient and his/her family (making and maintaining tactile contact with a patient), and specific nursing interventions in ICU dependent on patient's status (application of thermoregulatory procedures).

The most frequently repeated activities which were not considered in the TISS-28 scale were the interventions within “assessment of patient's health status,” performed 533 times, and the average duration of these interventions was 68.39 sec (Me = 19, SD = 213.68). The total time devoted to the performance of these activities was 36455.43 sec and constituted 8.85% of the total nursing time during the period examined. The most troublesome activities within this scope of interventions were direct and indirect observations (monitors).

During the period of the study, occurred interventions within “making and maintaining therapeutic contact with a patient and his/her family” 243 times. The mean duration of an intervention within these activities was 67.60 sec (Me = 42, SD = 72.17). The total amount of time devoted by nurses to making and maintaining contact with a patient or his/her family was 16427.9 sec, which constituted 3.99% of the nursing time.

Activities associated with “assistance with personal hygiene” were observed 148 times. However, compared to the activities within the 2 previously mentioned scopes, the mean duration of these interventions was considerably longer—399.26 sec (Me = 250, SD = 466.78). In general, the nurses needed 59091.8 sec for performing these tasks, which was 14.36% of the nursing time. The most time consuming intervention was toilet of patient's whole body, for the performance of which each time the nurses devoted 784,94 sec per one patient on average.

Activities within “assistance to patients with nutrition” were noted 186 times during the study. The mean duration of these activities was 232.92 sec (Me = 189.2, SD = 229.54). The total time of nursing interventions in this area was 43323.9 sec which constituted 10.53% of the nursing time. The activity most often performed was feeding a patient that was 158 times, which required from a nurse the saving of 252.96 sec per one patient on average.

Considering the activities within “maintenance of patency of catheters” the interventions not included in the TISS-28 scale were repeated 75 times during the study; their mean duration was 62.2 sec (Me = 15, SD = 135.65). In general, the nurses needed 4665 sec for performing these activities, which constituted 1.13% of time devoted to nursing during the period analyzed. The intervention that was most often repeated by nurses within this scope of activities was flushing the peripheral venous catheter 64 times, in which they needed 21.26 sec on average.

The interventions within “assistance to patient with excretion” occurred 24 time, and their mean duration was 56.90 sec (Me = 25, SD = 79.31), a total of 1365.8 sec, which was 0.33% of the nursing time. The highest activity within this scope of interventions was associated with providing a patient with a bedpan.

During the period analysed, the activities concerning “assistance to patient with maintaining physical activity” were repeated 14 times. The mean duration of these activities was 139.98 sec (Me = 124.1, SD = 88.89), and the total amount of time devoted by nurses for performing these activities was 1959.8 sec, which was 0.48% of the nursing time.

In the category “specific nursing interventions in the ICU dependent on patient's status,” activities associated with the process of thermoregulation of the organism were observed 17 times. These interventions lasted for 43.78 sec. on average, and the total time of their performance was 744.3 sec—0.18% of time devoted to nursing in the intensive care units in the study.

Among the activities not considered in the TISS-28 scale, the greatest workload among nurses resulted from hygienic activities—14.36% of working time and from assistance to patients with nutrition—10.53%. In the units examined, much attention was attached to the evaluation of the health status of a patient—8.85% of the nursing time. The remaining areas required less attention from nurses. Maintaining the patency of catheters occupied 1.13% of the nursing time, while in the remaining areas this time did not exceed 0.5%. Based on the median test, a highly significant statistical difference was observed on the level of *χ* ^2^ = 177151, ***P < 0.001 between individual scopes of activities not considered in the original TISS-28 scale.

Table 2 presents the total number, total duration, and relation to the total nursing time of interventions within the categories in the TISS-28 scale, and scopes of activities, which were not considered in this scale.

Based on the median test, a highly significant statistical difference was noted on the level of *χ* ^2^ = 169458, ***P < 0.001 between the nurses' workload resulting from performance of activities qualified into the TISS-28 scale and load resulting from performance of interventions within the scopes of nursing care not considered in this scale.

## 4. Discussion

In many countries, the TISS-28 scale is adopted as a golden means enabling a rational management of human resources, especially the occupational group of nurses. Undoubtedly, it possesses several advantages, including the rational assessment of a patient, clarity, ease and speed of data collection. According to this scale, a well-trained nurse, while providing care for a patient requiring intensive care, may work out from 40–50 scores per shift, or in other terms, needs from 10 to 20 minutes for the provision of 1 score [6, 22]. Hariharan et al. [23] in their studies observed that nurses worked out 26.2 scores per day. Based on the studies conducted in Polish conditions, it was noted that a nurse is capable of working out 55 scores according to the TISS scale during one 12-hour shift [24]. The difference in the number of scores obtained by nurses in individual health care systems may be due to the differences in the severity of the patients' state. This element should not be of a great importance, assuming that as patients requiring intensive care are considered as those who obtain 20–39 according to the TISS-28 scale, whereas patients who obtain from 10–19 scores require intensive monitoring only, and do not have to be hospitalized in an ICU. Thus, the reports appearing in literature pertaining to the differences in the number of scores worked out between systems of health care in various countries must, therefore, be associated with the nursing itself.

The interventions mentioned in the TISS-28 scale focus mainly on diagnostic-treatment procedures within the scope of intensive care, generally omitting the activities associated with intensive nursing, especially those for which a Polish nurse is prepared during vocational education. In addition, in the national system of health care, there is no lower level in nursing teams, for example, nurse assistant, or nursing assistant. Abroad, these employees take over many activities associated with nursing interventions, which results in a considerable decrease in the workload among nurses employed in Western ICUs; however, it hinders the correct usage of this scale for the needs of nursing staff scheduling in Polish ICUs. The authors of the scale themselves noticed the risk of interferences resulting from cultural and organizational differences between individual systems of health care [25]. Other researchers have also emphasized this fact, indicating it as one of the factors causing differences in the evaluation of the demands for care based on the TISS-28 scale [26]. Nevertheless, there are no reports describing detailed disproportions between the original version of the TISS-28 scale and the local nursing practice.

In the case of Polish intensive care units, 75 interventions performed by nurses were observed. In the studies by other researchers, the number of activities mentioned was smaller than, for example, 45 [27]. Probably, the primary version of this scale—TISS-76—despite being more time-consuming, would to a greater degree reflect the engagement of Polish nurses in patient care, because it would contain a larger number of criteria [25]. In the TISS-28 scale, in scoring, the routine activities performed in Polish ICUs are not considered, for example, insertion of catheters, changing of dressings, or performance of ECG. These procedures do not affect the evaluation of patient's condition from the aspect of the severity of patient's state but are of great importance for the evaluation of the correct loading the nurses with work. This scale considers sole activity, for example, feeding by gastric tube, but does not take into consideration activities associated with its care, maintenance of patency, or prevention of complications. It is also noteworthy that certain score values evoke some reservations from the aspect of the actual contribution of Polish nurses in their performance. This refers in particular to the score value 1, ascribed to the activities within the scope of the category 1, referring to antibedsore prophylaxis. This scope of problems covers many activities, for example, evaluation of patient's exposure to the development of bedsores, changing patient's body position, facilitating blood flow, or skin care. The majority of these interventions are time-consuming and require systematic repetitions, for example, changing of body position. Such an attitude causes underestimation of workload among nurses and patient demand. Similar difficulties are also observed by researchers from other countries [28].

## 5. Conclusions

The TISS-28 scale allows evaluation of the state of health of patients hospitalized in ICU. This scale is also used for determination of nurse staffing based on patient demand for nursing care. Nevertheless, the presented results based on an example of Polish ICUs confirm that it cannot be approached as a golden means by the managerial staff planning nurse staffing in intensive care units. Nursing activities which are included in the scoring of this scale, and those which are not qualified into it, have been specified in detail. Due to this, practical implications of the results presented are possible, which allow a rational use of human resources in accordance with the actual scope of the activities undertaken. Using an original version of the scale, without consideration of the actual scope of the tasks performed, may lead, among other things, to a quicker development of the occupational burn-out syndrome among nurses or a decrease in the level of quality of nursing care. Therefore, the application of the TISS-28 for the needs of nursing staff scheduling in individual systems of health care should be preceded by a detailed analysis of cultural and organizational conditions in an individual system. The results obtained may also be a basis for the implementation into the intensive care units of medical caregivers, nursing assistant, as they indicate the area of care giving activities, which may be taken over from nurses by auxiliary staff. This may bring about measurable benefits from the economic aspect, resulting in the reduction in the costs of ICU functioning. The presented study may constitute an origin for further studies associated with the actual usefulness of the TISS-28 scale for nursing staff scheduling in individual systems of health care.

## Figures and Tables

**Table 1:** Frequency of occurrence and mean duration of observed interventions performed by Polish nurses in ICU.

No.	Intervention	*N*	Mean time of performance in sec.	Median (Me)	SD
1	2	3	4	5	6
1	Direct observation of a patient	513	34.28	18	60.37
2	Removing mucus from the airways	357	23.37	14	92.18
3	Measurement of arterial blood pressure	250	40.65	34	30.67
4	Making and maintaining contact with patient's family	229	67.91	40	73.70
5	Change of patient's body position	221	365.47	240	430.27
6	Measurement of diuresis	197	34.20	30	27.79
7	Performance of intravenous injection	196	66.21	42.6	57.89
8	Feeding of a patient	158	252.96	189.2	239.97
9	Connecting intravenous drip	152	88.98	81.5	65.07
10	Measurement of body temperature	103	16.89	6	21.92
11	Placing of pulsoxymeter	75	6.84	4.9	6.81
12	Taking venous blood for laboratory tests	69	92.94	50	102.22
13	Flushing of peripheral venous catheter	64	21.26	13.5	21.17
14	Changing dressing on wound	60	137.05	127	136.62
15	Removing of mucus from the oral cavity	58	45.00	24.5	46.21
16	Connecting infusion pump	58	80.41	62.5	61.77
17	Disconnecting infusion pump	52	47.51	36.5	50.21
18	Toilet of patient's whole body	52	784.94	578.3	580.63
19	Care of peripheral venous catheter	48	63.75	18	94.53
20	Measurement of saturation	47	38.65	30	29.43
21	Disconnecting intravenous drip	44	53.35	22.5	88.05
22	Performance of inhalation	43	434.04	409	154.74
23	Partial toilet of a patient	41	184.40	94.1	192.34
24	Changing bed sheets with a patient in bed	40	241.84	158.7	161.66
25	Deflation of tracheal tube cuff	35	18.82	18	10.74
26	Application of conveniences or immobilization of a patient	33	77.03	47	55.44
27	Administration of oral drug	31	26.70	20	27.80
28	Assisting at procedures and diagnostic tests	31	323.25	120	512.77
29	Administration of oxygen	22	15.59	9	13.75
30	Supporting expectoration of secretion from the airways	22	311.36	300	24.74
31	Exchange of nebulisator	20	14.53	14.1	7.07
32	Exchanging Redon's bag	20	63.25	60	14.53
33	Performance of subcutaneous injection	19	15.63	14	7.13
34	Provision of bed pad	18	64.98	25	89.62
35	Tapping patient's back	18	35.81	30.5	12.32
36	Performing thermoregulatory procedures	17	43.78	17.1	36.64
37	Insertion of gastric tube	16	151.62	173.5	88.91
38	Indirect observation of a patient	16	1165.62	1200	413.31
39	Feeding by gastric tube	16	358.12	360	16.31
40	Making tactile contact with patient	14	62.5	52	40.87
41	Connection of ECG electrodes	14	75	31.5	95.27
42	Carrying out of respiratory exercises	14	72.78	55	58.82
43	Suction of gastric tube	13	57.83	73	24.42
44	Changing tracheostomial tube tie	13	90.30	83	56.39
45	Administration of inhalation drugs	13	9.61	9	4.92
46	Flushing of gastric tube	13	165.76	180	47.69
47	Connecting hemodialysis	12	994.41	429	973.07
48	Care of sucking drainage	10	72.2	29	58.38
49	Change of dressing on central venous catheter	10	404	235	356.81
50	Hygienic washing of hands	10	82	60	94.46
51	Rehabilitation exercises	10	110.28	124.1	29.23
52	Removal of gastric tube	7	15.71	16	7.47
53	Disconnecting hemodialysis	7	608.71	430	433.01
54	Connecting blood or blood-related products	7	131.57	150	53.73
55	Insertion of peripheral catheter	6	439.33	332.5	218.46
56	Removal of peripheral catheter	5	133.6	62	164.87
57	Application of drug on skin	5	102	102	0
58	Flushing of sucking drainage	5	50	50	0
59	Administration of oral fluids	5	164	138	126.53
60	Provision of a strip of lignin	5	44	44	0
61	Application of ointment into nose	5	67.8	30	65.20
62	Application of suppository	5	9.2	10	3.11
63	Taking smear for laboratory tests	4*	54.5*	69*	29*
64	Tilting a patient to erect position	4*	214.25*	192.5*	146.23
65	Performance of intramuscular injection*	3*	26.66*	22*	11.71*
66	Exchanging urine bag	3*	11.66*	10*	2.88*
67	Flushing ultrafiltrator's filter	3*	683.33	655	71.82
68	Taking capillary blood for laboratory tests	2*	6.5*	6.5*	2.12*
69	Performance of enema	2*	64.5*	64.5*	6.36*
70	Application of ointment on eye	2*	340*	340*	0*
71	Measurement of central venous pressure	1*	138*	138*	—
72	Ventilation with Ambu bag	1*	28*	28*	—
73	Removal of urinary catheter	1*	32*	32*	—
74	Performance of ECG	1*	738*	738*	—
75	Performance of intradermal injection*	1*	9*	9*	—

Total number of time schedule measurements of duration of nursing interventions		3697			

*N*: number of measurements of an individual intervention.

*number of measurements smaller than 5.

**Table 2:** Total structure of loading nurses with activities considered and not considered in the TISS-28 scale.

Interventions	Total duration in sec.	No. of measurements	Mean	Standard deviation	% of nursing time
TISS-28 scale					
Basic procedures	163280.1	1562	104.53	42.6	39.67
Respiratory therapy	40627.5	603	67.37	142.91	9.87
Cardiovascular therapy	10021	31	323.25	512.77	2.44
Renal therapy	24981.8	219	114.07	338.26	6.07
Metabolic treatment	8636.8	42	205.63	132.07	2.1
Total	**247547.2**	**2457**	**100.75**	**217.26**	**60.15**
Not qualified into the TISS-28 scale					
Assistance with personal hygiene	59091.8	148	399.26	466.78	14.36
Assistance to patients with nutrition	43323.9	186	232.92	229.54	10.53
Evaluation of patient's health status	36455.43	533	68.39	213.68	8.85
Making and maintaining therapeutic contact	16427.9	243	67.60	72.17	3.99
Maintaining patency of catheters	4665	75	62.2	135.65	1.13
Assistance to patient with maintaining physical activity	1959.8	14	139.98	88.89	0.48
Assistance to patient with excretion	1365.8	24	51.25	76.35	0.33
Specific nursing activities in ICU dependent on patient's status	744.3	17	43.78	36.64	0.18
Total	**164033.9**	**1240**	**133.17**	**140.00**	**39.85**

Total	411581.13	3697	144.52	155.71	100

*χ*² = 16945.8; ***P < 0.001.
